# The Anti-Proliferative and Anti-Invasive Effect of Leaf Extracts of Blueberry Plants Treated with Methyl Jasmonate on Human Gastric Cancer In Vitro Is Related to Their Antioxidant Properties

**DOI:** 10.3390/antiox9010045

**Published:** 2020-01-04

**Authors:** Alejandra Ribera-Fonseca, Danae Jiménez, Pamela Leal, Ismael Riquelme, Juan Carlos Roa, Miren Alberdi, Richard M. Peek, Marjorie Reyes-Díaz

**Affiliations:** 1 Centro de Fruticultura, Facultad de Ciencias Agropecuarias y Forestales, Universidad de La Frontera, Avenida Francisco Salazar 01145, P.O. Box 54-D, Temuco 4811230, Chile; 2 Center of Plant-Soil Interaction and Natural Resources Biotechnology, Scientific and Technological Bioresource Nucleus (BIOREN-UFRO), Universidad de La Frontera, Avenida Francisco Salazar, P.O. Box 54-D, Temuco 4811230, Chile; danae.jimenez.carcamo@gmail.com (D.J.); miren.alberdi@ufrontera.cl (M.A.); 3 Center of Excellence in Translational Medicine (CEMT), Biomedicine and Translational Research Laboratory, Universidad de La Frontera, Avenida Alemania 0458, 4th Floor, P.O. Box 54-D, Temuco 4810296, Chile; pamela.leal@ufrontera.cl; 4 Instituto de Ciencias Biomédicas, Facultad de Ciencias de la Salud, Universidad Autónoma de Chile, Temuco 4180101, Chile; ismael.riquelme@uautonoma.cl; 5 Department of Pathology, UC Centre for Investigational Oncology (CITO), Advanced Centre for Chronic Diseases (ACCDis), The Millennium Institute on Immunology and Immunotherapy, Pontificia Universidad Católica de Chile, Santiago 8330034, Chile; jcroa@med.puc.cl; 6 Division of Gastroenterology, Department of Medicine, Vanderbilt University Medical Center, Nashville, TN 37232-0252, USA; richard.peek@vanderbilt.edu; 7 Department of Pathology, Microbiology, and Immunology, Vanderbilt University Medical Center, Nashville, TN 37232-0252, USA; 8 Departamento de Ciencias Químicas y Recursos Naturales, Facultad de Ingeniería y Ciencias, Universidad de La Frontera, Avenida Francisco Salazar, P.O. Box 54-D, Temuco 4811230, Chile

**Keywords:** gastric cancer, methyl jasmonate, antioxidants, anticarcinogenic, blueberry, phenols

## Abstract

Gastric cancer is the third main cause of cancerous tumors in humans in Chile. It is well-accepted that a diet rich in antioxidant plants could help in fighting cancer. Blueberry is a fruit crop with a high content of antioxidants. Methyl jasmonate (MeJA) is a phytohormone involved in plant defenses under stress conditions. The exogenous application of MeJA can improve the antioxidant properties in plants. We studied in vitro and in vivo anticancer action on human gastric cancer (cell line AGS) and the antioxidant properties of extracts from blueberry plants untreated and treated with MeJA. The results demonstrated that leaf extracts displayed a higher inhibition of cancer cell viability as well as greater antioxidant properties compared to fruit extracts. Besides, MeJA applications to plants improved the antioxidant properties of leaf extracts (mainly anthocyanins), increasing their inhibition levels on cell viability and migration. It is noteworthy that leaf extract from MeJA-treated plants significantly decreased cancer cell migration and expression of gastric cancer-related proteins, mainly related to the mitogen-activating protein kinase (MAPK) pathway. Interestingly, in all cases the anticancer and antioxidant properties of leaf extracts were strongly related. Despite highlighted outcomes, in vivo results did not indicate significant differences in *Helicobacter pylori* colonization nor inflammation levels in Mongolian gerbils unfed and fed with blueberry leaf extract. Our findings demonstrated that MeJA increased antioxidant compounds, mainly anthocyanins, and decreased the viability and migration capacity of AGS cells. In addition, leaf extracts from MeJA-treated plants were also able to decrease the expression of gastric cancer-related proteins. Our outcomes also revealed that the anthocyanin-rich fraction of blueberry leaf extracts showed higher in vitro antiproliferative and anti-invasive effects than the crude leaf extracts. However, it is still uncertain whether the leaf extracts rich in anthocyanins of blueberry plants are capable of exerting a chemopreventive or chemoprotective effect against gastric cancer on an in vivo model.

## 1. Introduction

Gastric cancer is the leading cause of cancer-related death in Chile and the second worldwide, mainly in older males [1,2]. Despite the advances in treatments of this pathology and the decrease of precursor lesions [3], recent reports suggest a likely increase in gastric cancer mortality, particularly in young people in both Eastern and Western countries [4]. These outcomes, together with the predictions of global mortality for the next 20 years, warn that gastric cancer will be the tenth leading cause of death overall within the following 25 years [5]. According to Wu and Chung [6], the high rate of mortality associated with gastric cancer is due to its early stages which are asymptomatic; this hinders detection and subsequent treatment of this type of cancer.

Gastric inflammation related to gastric cancer development in humans can be defined as a highly complex biochemical protective response to cellular/tissue injury induced by oxidative stress. *Helicobacter pylori* infection and the ingestion of non-steroidal anti-inflammatory drugs (NSAIDs) are recognized as the major factors that cause oxidative injury to gastric mucosa in humans [7,8,9]. During the host colonization process, *H. pylori* induces a strong inflammatory response, generating large amounts of reactive oxygen species (ROS) [9,10,11]. Besides, various reports indicate that several risk factors such as ethanol exposition, smoking, and diets high in salt and fat, which induce the generation of reactive oxygen species, may trigger human gastric cancer due to the ROS-induced oxidative damage to the gastric mucosa [12].

Oxidative stress is a state in which toxic reactive oxygen species overcome the endogenous antioxidant defense in the host [13,14,15]. Therefore, it is well-accepted that the damage caused by ROS in human health can be counteracted by a diet with fruits and vegetables rich in antioxidants, thus reducing the risk of gastric cancer progress [4,16,17]. Berry plant species are recognized as good sources of antioxidants, with a wide range of chemical compounds, mainly anthocyanins. They reduce effectively the risk of chronic and degenerative diseases, including cancer [18,19,20]. Despite the promising anticarcinogenic properties of blueberries [21], mainly anthocyanins, it is reported that they have a low bioavailability compared with other phenolic compounds [22,23].

Several studies have shown that the healthy properties of fruit species can be affected by a series of factors such as genetic background, environmental conditions, cultural practices, and post-harvest handling. Earlier studies showed that both pre- and post-harvest applications of the volatile signal molecule methyl jasmonate (MeJA) induce the synthesis of phenolic compounds with antioxidant properties in berries [24,25,26,27], improving their potential advantageous impacts on human health.

The present work was aimed to assess the anticancer effects against human gastric cancer as well as antioxidant properties of extracts from highbush blueberry plants untreated and treated with MeJA.

## 2. Materials and Methods

### 2.1. Blueberry Growth Condition and Pre-Harvest MeJA Treatment

Six-year-old plants of highbush blueberries (*Vaccinium corymbosum* L., cultivar Legacy) grown at Berries San Luis Farm, Lautaro, Chile, both untreated and treated with methyl jasmonate (MeJA), were used to obtain the leaf extracts. All plants were maintained under conventional agronomic management practices. For MeJA treatment applications, an aqueous solution (distilled water supplemented with Tween 80 at 0.05% *v*/*v*) at a final concentration of 0.05 mM of MeJA was sprayed (moisturizing area of 100 mL per bush) to the above-ground part of the blueberry bushes, in a single application, at the phenological stage of the fruit set. Distilled water plus Tween 80 at 0.05% *v*/*v* was sprayed to MeJA untreated plants. The MeJA dose was selected in accordance with previous studies performed by the research group. An experimental randomized block design was used, with five blocks per treatment with 10 bushes each. All foliar sprays were performed the same day, early in the morning, using a back-held spray pump. For extract preparation, fully-expanded leaves or fresh mature fruits were collected at the phenological stage of fruit harvest.

### 2.2. Preparation of Blueberry Aqueous Crude Extracts

Crude extracts were obtained by macerating 10 g of fresh leaves or fresh fruits with 100 mL of ultrapure water in a chilled (4 °C) mortar. Then, the extracts were centrifuged at 5000 rpm for 5 min, and the supernatants were collected. The pellet was subjected to three sequential extractions, every time using an ultrapure water volume of 100 mL. All the supernatants were mixed in order to obtain the final crude extract, which was filtered and lyophilized to dryness. Prior to use, the dried extracts were stored at 4 °C in the dark.

### 2.3. Antioxidant Properties of Crude Extracts

The activity of free radical removal of the extracts was determined using the method of 2,2-diphenyl radical-1-picrylhydrazyl (DPPH), as described by Chinnici et al. [28]. Absorbance was measured at 515 nm and the antioxidant activity was expressed as Trolox Equivalents (TE). Total phenol content was assessed at 765 nm by the Folin–Ciocalteu method, according to Slinkard and Singleton [29], using chlorogenic acid (CA) as standard. Total flavonoids were measured by the colorimetric method described by Zhishen et al. [30], using rutin as standard. Briefly, the extracts were measured at 595 nm in a UV-visible spectrum (UV-VIS Spectrophotometer T80+, PG Instruments, Beijing, China) and the results were expressed in equivalents of rutin. Finally, total anthocyanins were analyzed with the method described by Cheng and Breen [31]. For this purpose, the absorbance was determined in a spectrophotometer at 530, and 657 nm, and the results were expressed as cyanidin 3-glucoside (c3g) equivalents.

Phenol composition of blueberry leaf extracts was analyzed by high-performance liquid chromatography (HPLC), using a HPLC system (model LC-Net II/ADC, Jasco, Tokyo, Japan)) equipped with a detector diode array (DAD, model MD 2015 Plus, Jasco, Tokyo, Japan) and a reverse-phase column (RP-18, Kromasil 250 × 4.6 mm; Waters, Milford, MA, USA).). Phenolic acids and flavonoids were analyzed according to the method proposed by Ruhland and Day (2000), modified [32] with a flow rate of 1.0 mL min^−1^. The phenolic caffeic, ferulic, *p*-coumaric, and chlorogenic acids, and the flavonoids quercetin, myricetin, kaempferol, and rutin were used as standards (Sigma Chemical Co., St. Louis, MO, USA). Signals were detected at 320 nm. Acidified water (phosphoric acid 10%) (A) and 100% acetonitrile (B) composed the mobile phase. The eluent gradient was: 0–9 min of 100% A, 9.1–19.9 min of 81% A and 19% B, and 20–25 min of 100% B. Besides, the anthocyanidin profile was analyzed by HPLC using the method based on anthocyanidin (anthocyanin aglycone) determination, earlier described by Nyman and Kumpulainen [33], with minor modifications [32]. For the analyses, 0.3 g of whole fruits were extracted with 3 mL of acidified ethanol (pH 1) using a mortar and pestle at 4 °C. Then, the extracts were placed into centrifugation tubes, sealed, and boiled at 95 °C in a water bath during 1 h for the acid anthocyanin hydrolysis and incubated at 4 °C in the dark for 24 h. Finally, the extracts were centrifuged at 3000 rpm for 10 min and stored at 4 °C in the dark prior use. Signals were detected in the supernatants at 530 nm. Acidified water (acetic acid 10% *v*/*v*) (A) and absolute acetonitrile (B) were used as mobile phase with the following eluent gradient: 0–23.9 min of 90% A–10% B, 23.9–24.1 min of 80% A–20% B, 24.1–27 min of 20% A–80% B, and 27.1–37 min of 90% A–10% B. Delphinidin, malvidin, petunidin, cyanidin, and peonidin (Sigma Chemical Co., St. Louis, MO, USA) were used as standards.

### 2.4. Assessment of the Effect of Extracts on Gastric Cancer Cells Viability and Migratory Capacity

As a first step, we assessed the effect of leaves and fruit crude extracts from blueberry plants untreated with MeJA on the viability of gastric cancer cells, in order to detect the minimal effective doses. Work solutions were prepared with 100 mg of dried extract; dissolved in 100 µL of Dulbecco’s Phosphate-Buffered Saline (DPBS) buffer 1× (Thermo Scientific Hyclone, Logan, Utah, USA) and then diluted in Roswell Park Memorial Institute (RPMI) 1640 medium to reach final concentrations of 0, 100, 200, 400, 800, 1600, and 3200 µg mL^−1^. As a second step, we assessed the anticancer effect (cell viability and migration, and cancer-related protein expression) of leaf extracts from plants treated with MeJA at various concentrations.

Gastric cancer cells (line AGS) were grown in RPMI 1640 medium supplemented with 10% fetal bovine serum (FBS) and 1% penicillin/streptomycin (Thermo Scientific Hyclone, Logan, UT, USA) and incubated at 37 °C, 5% CO_2_, and 95% humidity.

Cell viability was determined by the AQueousCellTiter 96^®^ Non-Radioactive Cell Proliferation Test, according to the manufacturer’s recommendations (Promega Corp., Madison, WI, USA). Briefly, AGS cells were seeded (using three replicates) in 96-well plates at a final density of 3.5 × 103 cells/100 µL^−1^ of culture medium. After a cell adhesion period (24 h), cells were treated with the different extracts at the final concentrations mentioned above. At the end of 24 or 48 h of extract exposure, 20 uL of 3-(4,5,dimethylthiazol-2-yl)-5-(3-carboxymethoxy-phenyl)-2-(4-sulfophenyl)-2H-tetrazolium inner salt (MTS) reactive were added to each well. The plates were incubated during one hour at 37 °C, and the absorbance was determined at 490 nm using a plate reader PHOmo (Autobio Labtec Instruments Co. Ltd.,Zhengzhou Henan, China). The percentage of cell viability was calculated using the formula: Cell viability (%) = (X × 100%)/Y, where “X” is the absorbance of treated cells and “Y” the absorbance of untreated cells.

Cell migration was evaluated using 96-well chambers of 8-µm pores size (BD Biosciences, Bedford, MA, USA). Briefly, AGS cells were seeded in the chamber at a final density of 50 × 103, using 500 µL of culture medium (free of serum) and added at the bottom of the chamber using 750 uL of culture medium supplemented with 10% of fetal bovine serum. After the cell adhesion period (24 h), cells were treated with the extracts at the final concentrations described above and incubated at 37 °C during one hour. Subsequently, the culture medium containing the extract was removed and changed by a fresh serum-free culture medium. After 48 h of incubation, cells on the upper surface of the membrane were removed using a cotton swab. The cells capable of migrating to the lower compartment of the filters were fixed and stained with a solution of 0.1% crystal violet and 4% formalin. At least four random fields were analyzed to determine the migrated cell number by using an optical microscope (20× objective). All samples were evaluated in duplicate.

### 2.5. Assessment of the Effect of Leaves Extracts on Gastric Cancer-Related Protein Expression

We assessed the effect of blueberry leaf crude extracts on the expression of a protein related to the Protein Kinase B / mammalian target of rapamycin (AKT/mTOR) signaling pathway. Protein extraction was carried out using 1 mL of Radioimmunoprecipitation Assay RIPA buffer (150 Mm NaCl, 1.0% IGEPAL CA-630, 0.5% sodium deoxycholate, 0.1% SDS, 50 mM Buffer Tris-HCl, pH 8.0; Sigma-Aldrich, Merck KGaA, Darmstadt, Germany), supplemented with 60 uL of phenylmethylsulfonyl fluoride (PMSF, Sigma-Aldrich) and 10 mL of Phosphatase Inhibitor Cocktail 2 (Sigma-Aldrich). For the protein inhibition assay, AGS cells were seeded in 10 cm^2^ culture plates at a final density of 1 × 10^4^ cells to reach 80% of confluence after a 24-h starvation period. Then, cells were treated with the extracts at different concentrations indicated above and incubated at 37 °C for 2 h. A free culture medium supplemented with serum DPBS buffer was used as the control treatment. After extract treatments, cells were washed three times with cold DPBS and ice-stored (vortexing occasionally) for 30 min. Then, cells were lysed using 750 mL of RIPA buffer, and the sonicated cell lysate was centrifuged at 10,000 rpm at 4 °C for 15 min. Proteins quantification was performed by the BCA assay (Thermo Fisher Scientific, Waltham, Massachusetts, USA). For the Western Blot assay, 40 µg protein were used, which were separated by polyacrylamide gel electrophoresis NuPAGENovex 4–12% Bis-Tris (Invitrogen, Thermo Fisher Scientific, Waltham, MA, USA) and transferred to Immobilon™-P PVDF membrane (Sigma-Aldrich) according to the manufacturer’s recommendations. Subsequently, membranes were blocked (1 h at room temperature with 5% milk-TBST) and incubated with the primary antibodies (Cell Signaling Technology Inc., Danvers, MA, USA; see Table 1).

### 2.6. Obtainment of a Rich-Anthocyanin Extract from Blueberry Leaves

Rich-anthocyanin extracts of blueberry leaves from plants untreated and treated with MeJA were obtained through a column chromatography fractionation of aqueous crude extracts using a glass column of 2.5 cm in diameter. Briefly, a cotton pad was put at the end of the column, which was packed with silica gel previously dissolved in dichloromethane to reach 12 cm in height. Then, the lyophilized extract of leaves (1 g) was dissolved in dichloromethane and mixed with 1.5 g of silica gel (previously dissolved in dichloromethane) to obtain a smooth paste, which was placed at the top of the column with a filter paper. The column was eluted with 1 L of ethyl acetate, and then with 1 L of acidified ethanol pH 1.0 (0.82 mL of hydrochloric acid (HCl, 37%) to 100 mL of absolute ethanol). Finally, the solvent was rotary-evaporated to dryness (Heidolph VV2020) in order to obtain the dried anthocyanin-rich extract. The in vitro anticancer effects (inhibition of cell viability and migration) caused by these extracts, at a final concentration of 100 µg mL^−1^, was evaluated as described above (Section 2.4).

### 2.7. Study of the In Vivo Anticancer Effect of Leaves Extracts Using a Gerbil Model

The experimental protocol and treatments with gerbils were approved by the Institutional Animal Care and Use Committee (IACUC) from the USA (M/06/406). The work solution was prepared using the anthocyanin-rich extract from MeJA-treated plants dissolved in distilled water, considering an estimated daily water consumption of 7 mL for a total of 14 Mongolian gerbils. The extract solution was supplied to the beverage bottles containing drinking water, in order to allow a daily consumption of 200 mg of blueberry extract per gerbil kg (gerbil weight of 70 g). The preparation of the extract solution was performed daily in order to avoid both bacterial contaminations as well as extract oxidation in response to light exposure. In the same way, beverage bottles were covered with aluminum foil to prevent light exposure of blueberry extracts during treatment.

Gerbils were infected (challenged) with 5 × 10^9^ colony forming units (CFU) of *H. pylori* (highly carcinogenic strain 7.13) dissolved in 0.5 to 1 mL of *Brucella* broth, which was supplied by orogastric feeding needle with rubber cap. The animals were infected twice with a difference between 48 h each time (day one and day 39) to confirm stomach infection. Fed *Brucella* broth and *H. pylori* untreated gerbils were used as a negative control treatment.

As a first step, we evaluated the gastric cancer chemopreventive effect, which is the ability of blueberry extracts to prevent the infection of *H. pylori* in gerbils. An eight-gerbil group (Group 1) was treated with a normal meal (absolute control) or blueberry extract at a final concentration of 2 mg mL^−1^ (ad libitum) during two weeks prior to the infection with *H. pylori*. After infection, the gerbils were maintained during eight weeks with a diet based on water and normal meal and then sacrificed using CO_2_ in order to evaluate the effect of treatments on the gastric mucosa. As a control treatment, a second eight-gerbil group (Group 2) was treated with regular food and water for two weeks, prior to infection with a strain of *H. pylori*. After infection, the gerbils were maintained for eight weeks with water and normal meal and then sacrificed using CO_2_ to analyze the effect of this control treatment in the gastric mucosa.

As a second step, we evaluated the chemoprotective action (to prevent the progression of *H. pylori* infection in gerbils) of blueberry extracts. A third eight-gerbil group (Group 3), after being infected with an *H. pylori*, was treated with the blueberry extract (supplied as drinking water, ad libitum, at a final concentration of 2 mg mL^−1^) or with the normal feed and water during two weeks. After infection, and before blueberry extract exposure, infected gerbils were maintained under normal feeding during four weeks in order to ensure both the *H. pylori* adhesion to the gastric mucosa as well as lesion development. After the treatment with the extract, gerbils were fed for eight weeks with normal meal and water. Then, gerbils were sacrificed using CO_2_ to evaluate the effect of the extract in the gastric mucosa. As a control treatment, another eight-gerbil group (Group 4), prior to the infection with *H. pylori*, was fed with a normal diet for 14 weeks. After this time, gerbils were sacrificed using CO_2_, in order to evaluate the impact of treatments in the gastric mucosa.

Finally, we studied the safety of the blueberry extract in normal gastric mucosa. For this, a five-gerbil group (Group 5) was maintained with a normal diet during four weeks and exposed to a gastric dose of *Brucella* broth (culture medium without *H. pylori*). Then, they were treated with the blueberry extract (supplied as drinking water, ad libitum, at final concentration 2 mg mL^−1^) for two weeks. After eight weeks, gerbils were maintained under regular conditions (food and water) and then sacrificed using CO_2_ to evaluate the effect of the extract in the gastric mucosa. As control treatments, a five-gerbil group (Group 6) was exposed to gastric *Brucella* broth (culture medium without *H. pylori*) and then maintained under the normal food and water conditions for ten weeks. Then, gerbils were sacrificed using CO_2_ in order to analyze the effect of this treatment in the gastric mucosa.

### 2.8. Statistical Analysis

Statistical analysis was performed using SPSS version 17.0 (SPSS Inc., Chicago, IL, USA). Differences between groups (treated and untreated with MeJA) were analyzed by ANOVA (one-way or two-way depending on the assay) and the Tukey test (*p* < 0.05). All data presented in the graphs or tables correspond to at least three independent analytical replicates (mean ± standard error). Mann–Whitney U tests were used to determine statistical significance between gerbil groups.

## 3. Results

### 3.1. In Vitro Anticancer and Antioxidant Properties of Blueberry Crude Extracts on Gastric Cancer Cells

**Figure 1:**
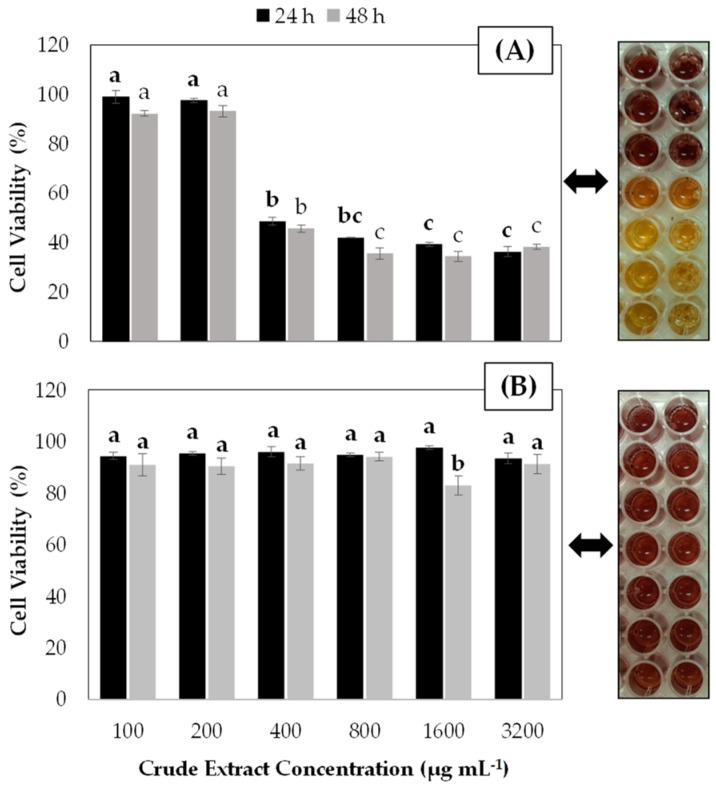
Effect of aqueous crude extracts of blueberry leaves (**A**) and fruits (**B**) on cell viability of the AGS gastric cancer cell line. Cell viability was measured after 24 and 48 h of extract incubation using the CellTiter 96^®^AQueous kit—One Solution Reagent assay. The percentage of viable cells was calculated in comparison to untreated control (water). Plotted values represent the mean of three independent experiments. Lower case letters (a–c) indicate significant differences among the treatments according to the Tukey test (*p* ≤ 0.05). Error bars represent the standard error of the data set. On the right side of each graph the coloring reaction of this assay is included, where discoloration indicates loss of viability.

Our results demonstrate that leaf extracts revealed a significantly higher inhibition of AGS cell viability than fruit extract (Figure 1). The inhibitory effect was dose-dependent. Leaf extracts at a final concentration equal or higher than 400 µg mL^−1^ caused more than 50% of AGS cell viability inhibition, reaching up to 60% of inhibition at the highest doses. There were no detected significant differences between incubation times evaluated (24 and 48 h) and cell viability.

It is noteworthy that leaf crude extracts exhibited higher antioxidant activity (up to 17.7-fold), total phenols (up to 13-fold), and total anthocyanins (up to 3.6-fold), compared to fruit extracts (Table S1).

### 3.2. Anticarcinogenic and Antioxidant Properties of Leaf Extract from Blueberry Plants Treated with MeJA

**Figure 2:**
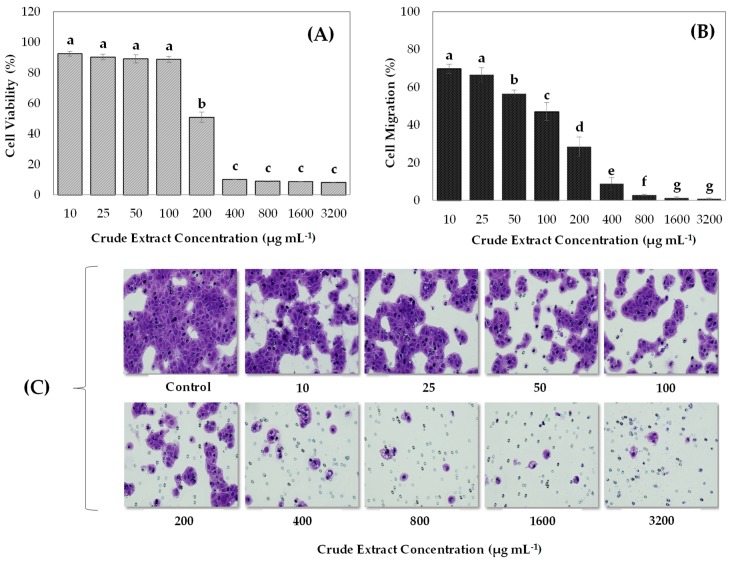
Effect of aqueous leaf crude extracts of blueberry plants treated with methyl jasmonate (MeJA) on cell viability (**A**) and cell migration (**B**) of the AGS gastric cancer cell line. Cell viability was measured after 24 h of extract incubation using AlamarBlue reagent and determining the fluorescence intensity at 530/560 nm. The percentage of viable cells relative to the control cells was calculated. To assess the migratory capacity, cells were exposed to increasing concentrations of leaf extracts during 1 h. The number of cells migrating in the presence of serum-free medium and Dulbecco’s Phosphate-Buffered Saline (DPBS) buffer represented 100% of cell migration (control), whereas the values of cell migration after the extract exposure were calculated in relation to the control. Lower case letters (a–g) indicate significant differences among the treatments according to the Tukey test (*p* ≤ 0.05). Error bars represent the standard error of the data set. (**C**) Representative images of the effect of each treatment on AGS cell migration under the light microscope and 20× objective.

As shown in Figure 2A, cell viability of AGS cells gradually decreased in response to increasing doses of leaf extract from MeJA treated plants. The inhibition levels reached up to 50% at a final concentration of 200 µg mL^−1^, with a greater impact (~92%) detected with doses of 400 µg mL^−1^ onward. Additionally, our findings indicated that the leaf extracts of MeJA-treated plants, at all concentrations studied, were able to decrease (in a dose-dependent way) the migratory capacity of AGS cells (Figure 2B,C). The extract was able to inhibit by 30% the number of migrated cells at a concentration of 10 µg mL^−1^ while at doses of 100 µg mL^−1^ the inhibition of cell migration reached up to 50%. Interestingly, extract at a concentration equal or higher than 200 µg mL^−1^ caused reductions in AGS migration ability of 70%, reaching 99% at the highest dose (3200 µg mL^−1^).

**Figure 3:**
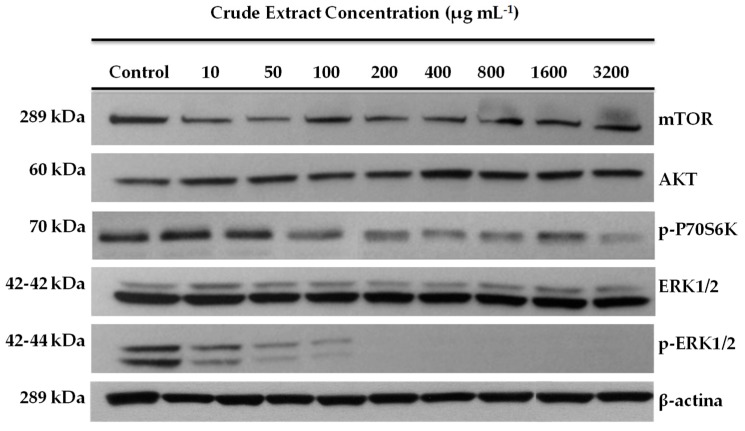
Effect of aqueous crude leaf extracts of blueberry plants treated with MeJA on the expression of gastric cancer-related protein in the AGS cell line. The inhibitory effect on protein expression of AKT/mTOR pathway was assessed by Western blot and 35–40 µg of protein was loaded in each well. The β-actin (45 kDa) was used as an internal loading control.

We also evaluated the effect of leaf extracts from MeJA-treated plants on the expression of proteins related to gastric cancer (AKT/mTOR and mitogen-activating protein kinases (MAPK) signaling pathways). Our results (Figure 3) showed that the total portions of each protein analyzed (mTOR and AKT) were not affected by the addition of increasing concentrations of blueberry extracts. Nonetheless, we observed almost a complete inhibition of the phosphorylated portion of the protein ERK1/2 (p-ERK1/2) in response to the application of the extract at a concentration higher than 100 µg mL^−1^. Moreover, we found that blueberry extracts at the same concentrations slightly decreased the expression of the phospho-P70S6Kprotein (Figure 3).

With respect to antioxidant properties of leaf extract from plants untreated and treated with MeJA, we found that MeJA-treated plants exhibited significantly higher antioxidant activity (up to 200%), total phenols (up to 78%), total flavonoids (up to 40%), and total anthocyanins (up to 63%) as compared to extracts from MeJA-untreated plants (Table 2).

Moreover, HPLC-DAD results (Table 3) revealed that phenolic acids did not vary between extracts from plants untreated and treated with MeJA. In contrast, according to flavonoid analysis, rutin and myricetin displayed a significant increment (up to 22% and 155%, respectively) in leaves of MeJA-treated plants compared to those detected in MeJA-untreated ones. Also, we found that leaf extracts from MeJA treated plants exhibited significantly higher concentrations of all the anthocyanidin molecules evaluated here (Table 4), including delphinidin (up to 27%), malvidin (up to 24%), cyanidin (up to 32%), petunidin (up to 33%), and peonidin (up to 17%).

Despite these outcomes, HPLC/MS/MS analysis showed no significant differences in the phenolic profile between extracts from MeJA-treated and -untreated plants (Table 5), except the compound myricetin-3-xylose, which only was detected in leaf extracts of MeJA-untreated plants.

### 3.3. Comparing the In Vitro Anticancer Effect between Crude and Rich-Anthocyanin Leaves Extracts

**Figure 4:**
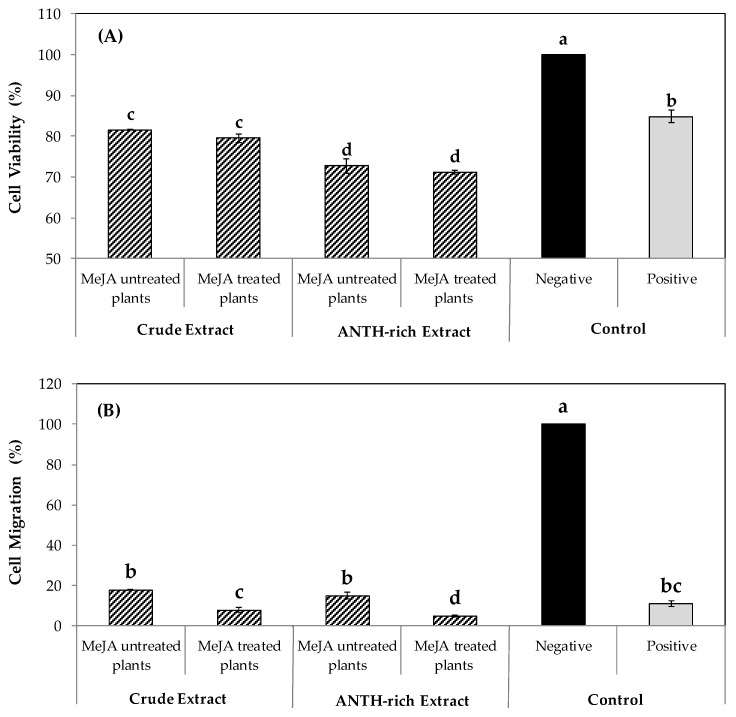
Effect of crude and anthocyanin-rich (ANTH-rich) extracts of leaves from plants untreated and treated with MeJA on cell viability (**A**) and cell migration (**B**) of the AGS gastric cancer cell line. Cell viability was measured after 24 h of extract incubation using AlamarBlue reagent and determining the fluorescence intensity at 530/560 nm. The percentage of viable cells relative to the control cells was calculated. To assess the migratory capacity, cells were exposed to increasing concentrations of leaf extracts during 1 h. The number of cells migrating in the presence of serum-free medium and buffer DPBS represented 100% of cell migration (control), whereas the values of cell migration after the extract exposure were calculated in relation to the control. All extracts were evaluated at a final concentration of 100 µg mL^−1^. Distilled water was used as a negative control, whereas a quercetin aqueous solution (4 µM) was used as a positive control. Lower case letters (a–d) indicate significant differences among the treatments according to the Tukey test (*p* ≤ 0.05). Error bars represent the standard error of the data set.

Our outcomes regarding in vitro anticancer effect revealed that both crude and rich anthocyanin (ANTH-rich) extracts from plants untreated and treated with MeJA significantly decreased AGS cell viability by around 20–30% compared to the negative control (Figure 4A). Indeed, these inhibitory effects were significantly higher than those reached with the positive control (quercetin 4 µM), being always greater for the ANTH-rich extracts (Figure 4). Moreover, our results showed that all blueberry extracts significantly reduced the AGS cell migratory capacity (Figure 4B). Of note, the higher decreases (up to 90%) were detected for the anthocyanin-rich fraction obtained from plants treated with MeJA, which were even greater than those observed by the effect of positive control (~85%).

### 3.4. Evaluation of Rich Anthocyanin Extracts of Leaves from MeJA-Treated Plants Over In Vivo Models

**Figure 5:**
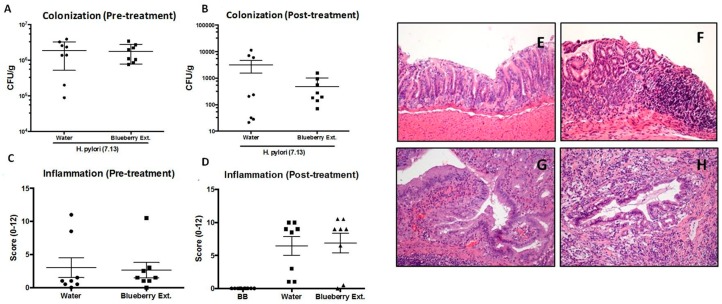
Effect of blueberry extracts enriched with anthocyanins over *H. pylori* colonization and injury on Mongolian gerbil gastric mucosa. (**Left**) Gastric inflammation 6–12 weeks after the challenge was assessed and scored (0 to 12) in uninfected (UI) and *H. pylori*-infected gerbils. Each data point represents the inflammation score from a single gerbil. Mean values are shown, and Mann–Whitney U tests were used to determine statistical significance between groups. (**Right**) Wild-type *H. pylori* strain 7.13 induces gastric inflammation, dysplasia, and adenocarcinoma in Mongolian gerbils. Hematoxylin and eosin–stained tissues (8 weeks after challenge) were evaluated for parameters of inflammation and injury by histopathology: Representative images of (**E**) uninfected gastric mucosa; (**F**) *H. pylori*-induced gastritis; (**G**) *H. pylori*-induced dysplasia; and (**H**) *H. pylori*-induced adenocarcinoma. Original magnification, ×200.

As described above, in order to evaluate the protective effect of blueberry anthocyanin-rich extract from MeJA treated plants over *H. pylori* colonization and injury to gastric mucosa, we used a Mongolian gerbil model. Gastric inflammation at 6–12 weeks after the challenge was assessed and scored (0 to 12) in uninfected and *H. pylori*-infected gerbils. Each data point represents the inflammation score from a single gerbil (Right, Figure 5). Wild-type *H. pylori* strain 7.13 induces gastric inflammation, dysplasia, and adenocarcinoma in Mongolian gerbils. Hematoxylin and eosin-stained tissues (eight weeks after challenge) were evaluated for parameters of inflammation and injury by histopathology Representative images of (E) uninfected gastric mucosa; (F) *H. pylori*-induced gastritis; (G) *H. pylori*-induced dysplasia; and (H) *H. pylori*-induced adenocarcinoma. Also, we examined the relation between inflammation and *H. pylori* colonization density in pre-treated and post-treated gerbils with blueberry extract (Left, Figure 5). No significant inverse relation was observed between the severity of gastric inflammation and *H. pylori* colonization density under both conditions.

## 4. Discussion

Gastric cancer is a global health pathology with a high rate of tumor incidence and mortality. Indeed, worldwide, it is the fourth most common cancer and the second highest cause of cancer-related mortality (1 million deaths per year) after lung cancer [34]. In Chile, this cancer is the leading reason for death from malignant tumors [1,2]. This pathology is commonly detected at advanced stages because in early stages it is clinically asymptomatic. For this reason it has a high mortality rate [6].

Oxidative stress is a state in which toxic ROS overcome the endogenous antioxidant defense of the host, which results in excess of free radicals that can react with cellular lipids, proteins, and nucleic acids, leading to cellular injury and eventual organ dysfunction [14]. It is well-known that the damage caused by ROS in human health can be counteracted by a diet rich in plant-derived antioxidants, which are capable of reducing the risk of cancer development [17]. Nowadays, significant scientific attention is focused on the role of antioxidant plants on the prevention and control of cancer, mostly by chemopreventive strategies [35,36]. Although conventional chemoprotective treatment methods, including surgery, chemotherapy, and radiation are dominant, the use of therapeutic dietary ways has gained importance in translational medicine [37].

Some fruits, such as berries, are recognized as important sources of vitamins, minerals, and antioxidant compounds of phenolic origin such as phenolic acids, flavonoids, and anthocyanins, which display high free radical scavenging capacity [32,38]. Moreover, several epidemiological studies have shown that human diets rich in fruits can reduce the risk of chronic and degenerative diseases like cancer [20,39]. In this regard, blueberries are a rich source of phenolic compounds, including chlorogenic acid, quercetin, kaempferol, rutin, myricetin, and mainly anthocyanins. They are recognized for their remarkable antioxidant properties [32,38] and as a useful option for counteracting human neurodegenerative diseases [40]. According to Jeyabalan et al. [41], blueberries are known as some of the most nutritious foods in the world, containing five major anthocyanidins: cyanidin, delphinidin, malvidin, peonidin, and petunidin. Interestingly, in blueberry species, anthocyanins are not only present in fruits but also leaves [38,42].

On the other hand, several reports highlighted that the healthy properties of fruits are affected by several factors including genotype, environmental conditions, agronomical management practices, and post-harvest handling. In this context and as was reviewed by Reyes-Diaz et al. [43], pre-and post-harvest applications of the signal molecule MeJA can induce the synthesis of healthy metabolites in some fruit crop species, thus improving their beneficial action on human health [26,44]. Considering these outcomes, the present study was aimed to assess the anticancer effect on human gastric cancer of extracts from blueberry plants (cv. Legacy) untreated and treated with MeJA in relation to their antioxidant properties. Our finding showed that leaf crude extracts displayed a higher inhibitory effect of AGS cell viability as well as better antioxidant properties compared to fruit extract. Therefore, we found that leaf crude extracts contained greater antioxidant activity as well as higher total phenols and anthocyanin concentrations than fruits. In accordance with these results, it has been reported that in different berries species, the total amount of phenolic compounds as well as total antioxidant activity are significantly higher in leaf tissues than in fruits [45,46]. Nonetheless, other studies showed that leaves and fruits have comparable capacities to scavenge free radicals [47].

Besides, we found that MeJA applications to blueberry plants increased (up to 30%) the inhibitory effect of leaf extracts on the viability of AGS cells. Also, significant increments on antioxidant properties (antioxidant activity, total phenolics, and total anthocyanins), were detected in blueberry plants treated with the phytohormone. Similarly, previous studies revealed that the application of MeJA on several fruit crops, via vapor, dipping or spraying, enhances their concentrations of antioxidant phenolic compounds and antioxidant activity. The induction of phenolic compound synthesis and/or accumulation of plant species in response to MeJA applications, including berries, was reviewed by Reyes-Diaz et al. [43]. In fact, there is evidence indicating that MeJA treatments increment anthocyanin accumulation in strawberries [48] and apple fruits [49]. Interestingly, Cocetta et al. [50] detected an increased expression of phenylalanine ammonium lyase, chalcone synthase, and anthocyanidin synthase genes in blueberry plants, as well as increments of epicatechin and anthocyanin concentrations in plants of blueberry treated with MeJA. In lettuce, the pre-harvest exogenous application of methyl jasmonate (MeJA) increased total phenolic compound content and antioxidant capacity, which was attributed to a caffeic acid derivative and an unknown phenolic compound [27]. In the same way, Li et al. [51] determined the optimal concentration of exogenous MeJA for the promotion of the sprouting in mung beans, showing that the application of this phytohormone resulted in improved antioxidant capacity and total polyphenols. The authors also found that MeJA promoted the production of polyphenols, metabolites, and antioxidants in the sprouts.

On the other hand, Seeram et al. [52] have shown that blackberry, raspberry, cranberry, red raspberry, strawberry, and blueberry extracts inhibit the growth of human oral, breast, colon, and prostate cancer cell lines in a dose-dependent manner. Additionally, juice of different small fruits, including highbush and lowbush blueberries, has been identified as strong antiproliferative and anti-inflammatory agents by inducing the apoptosis and cell cycle of the human stomach, prostate, intestine, and breast cancer cell lines [53]. According to Subash et al. [54], the protective effects of berry fruits on degenerative diseases are related to phytochemicals such as anthocyanin, caffeic acid, catechin, quercetin, kaempferol, and tannin. It is noteworthy that not only crude extract but also singly purified phenolic compounds from berry extracts can inhibit cancer cell proliferation, modulate cell cycle arrest, and induce apoptosis in cancer cells [19,55]. Indeed, results from in vitro and animal studies suggest that antioxidants from berry fruits, like anthocyanin and caffeic acid, have a beneficial role in neurodegenerative disorders due to their anti-inflammatory, anti-viral and anti-proliferative properties [56]. These beneficial effects have been attributed to their relatively high flavonoid content, in particular for anthocyanins [54]. Recently, when Demirel-Sezer et al. [57] were assessing the anticancer activity of blueberry (*Vaccinium myrtillis*), they found that the crude extract of this fruit caused a decrease of oxidant levels and oxidative stress index, and at an least 2-fold reduction in various apoptotic proteins was observed after quercetin and kaempferol (isolated from the crude extract) treatment. Furthermore, studies performed on various types of cancer cell lines demonstrated that blueberries also exhibit inherent abilities to prevent carcinogenesis, inhibiting the proliferation of neoplastic cells, which can reduce the risks of recurrence in patients in remission and could be helpful in the development of new strategies to treat cancer [58]. In the present research, flavonoids (rutin and myricetin) and almost all anthocyanidins displayed significant increments (range from 17% to 155%) in leaves of MeJA-treated plants compared to MeJA-untreated bushes, whereas phenolic acid composition did not vary. All phenolic compounds induced here by MeJA in blueberry leaves are recognized by their high antioxidant action [59], which could explain the high in vitro anticancer effect (cell viability and migration) on AGS gastric cancer cell in the extract of these leaves.

In addition, targets within AKT/mTOR and MAPK signaling pathways were analyzed due to their involvement in carcinogenic properties, including gastric cancer proliferation. We have shown that leaf extracts of MeJA-treated plants significantly decreased the expression of p-P70S6K (AKT/mTOR pathway) and p-ERK1/2 (MAPK) proteins. The activation by phosphorylation of P70S6K can induce the inactivation of 4E-BP1 protein, triggering the synthesis of a subpopulation of specific messenger RNAs that affects cell growth and homeostasis [60]. For this reason, p-P70S6K is considered as an effector molecule within the AKT/mTOR pathway. On the other hand, ERK1 and p-ERK1/2 proteins are well known to be activated in cancer, playing an important role in cancer cell proliferation, growth, and cell viability [61], either dependently or independently of the activation of AKT or mTOR proteins in the cells [62,63,64]. Our results suggest that the phosphorylation of p-P70S6K and ERK1/2 activates the abilities of cancer cells to remain constantly viable and resist apoptosis in order to perpetuate themselves over time. However, somehow the blueberry leaf extracts from plants treated with MeJA avoid this phosphorylation or increase the dephosphorylation of these proteins, reducing considerably their ability to remain viable in vitro. A similar conclusion can be obtained in relation to the characteristics of tumor migration/invasion since the activation of both pathways has been involved in the increase of migration/invasion in gastric cancer cell lines [65,66,67,68]. A future approach could be elucidate how blueberry extracts would be able to exert this effect on P70S6K and ERK1/2 in gastric cancer cells.

Dietary polyphenols exhibit several cellular effects by up- or down-regulating different key proteins related to signal transduction pathways, like cellular antioxidant defenses, cell-survival, and proliferation signaling through phosphoinositide 3-kinases (PI3Ks) and AKT, ERKs, or nuclear factor- enhancer of the chains kappa of activated B (NF-kB). Interestingly, cancer cell lines seem to be more sensitive to the anticarcinogenic effect of polyphenols than normal cells, due to the higher cytotoxicity of these molecules in cancer cells [69,70]. There are several ways whereby polyphenolic extracts may be decrease carcinogenic properties in cell lines. According to Costea et al. [71], polyphenols are able to hinder neoplastic processes such as proliferation, invasion, metastasis, or angiogenesis. Banerjee et al. [72] indicated that the ability of phenolics to down-regulate the pro-inflammatory enzymes and cyclooxygenase-2 (COX-2) and inducible nitric oxide synthase iNOS) is related to their prophylactic potentials as anticarcinogenic compounds. Moreover, it has been shown that flavonoids exert chemopreventive effects by acting at the level of protein kinase signaling pathway, then directly affect the phosphorylation state of proteins involved in cell signaling pathways related to the carcinogenesis processes [73]. Additionally, Kwon et al. [74] reported that phenolic molecules exhibit anti-inflammatory properties, inducing the detoxification of carcinogen enzymes (phase-II) and modulating subcellular signaling pathways of cancer cell proliferation, apoptosis, and tumor angiogenesis. According to Fuhrman et al. [75], plant phenols can avoid the susceptibility of plasma and low-density lipoprotein to lipid peroxidation through their antioxidant action. It is noteworthy that polyphenols may play both antioxidant and pro-oxidant roles; the antioxidant effect reduces the imbalance induced by the oxidative stress, and the pro-oxidant function induces cytotoxicity in cancer cells [71].

Anthocyanins are probably the largest group of phenols in the human diet, which have been used for several therapeutic purposes including the treatment of diabetic retinopathy, fibrocystic disease, and vision disorders [76,77]. They have also been used as chemoprotective agents [78]. Based on these antecedents, we compared the in vitro anticancer effects between crude extracts and the anthocyanin-rich extract obtained from leaves of blueberry plants, both treated and untreated with MeJA. Remarkably, our data revealed that the inhibition level on AGS cell viability was higher in the anthocyanin-rich extracts compared to crude extract (27% and 18%, respectively), independent of the MeJA treatments. With respect to AGS cell migration, crude and anthocyanin-rich extract from plants untreated with MeJA displayed comparable inhibitory effects; however, when plants were treated with the phytohormone, the inhibition levels of migratory capacity was higher in anthocyanin-rich extract (95%) compared to the crude extract.

It is important to highlight that many studies in different cell lines, animal models, and human epidemiological trials suggest a protective role of dietary polyphenols against different types of cancer. However, studies have included a systematic evaluation of the anticancer activity of phenolic compounds from blueberries in gastric cancer are very scarce. Therefore, as a last step, we assessed the potential in vivo anticancer properties (chemopreventive and chemoprotective effects) against inflammation and gastric mucosa injuries that could drive to gastric cancer of blueberry anthocyanin-rich using a model of a Mongolian *H. pylori* gerbil model infected (challenged) by a carcinogenic strain of *H. pylori* (7.13).

The Mongolian gerbil model that we used is currently considered the best model to assess the rapid development of gastric cancer caused by *H. pylori* infection. In this work, there were no significant differences in any of the variables evaluated in vivo; however, a slight tendency to decrease the degree of colonization by carcinogenic *H. pylori* 7.13 strain in the treatment with blueberry extract after a challenge by this bacterium was observed. The non-significant differences that were observed, beyond the low number of gerbils in each group, do not rule out the potential utility of blueberry extract. However, these results do make us reconsider other important factors to evaluate the antioxidant effect of cancer from animal models. For example, variables in antioxidants and animal models might have masked the real chemopreventive and anticancer effects of blueberry extract. It is known that most of the molecules with antioxidant function require a higher available dose and a longer exposure period to perform their effect in in vivo models compared to in vitro models [79]. The bioavailability of antioxidants is also an important point to be considered in these models. This could be determined by variables such as the chemical stability of the antioxidant in the administration solvent (water); the effective concentration of active ingredients of the available extracts in their administration scheme (ad libitum); interactions and biological activity of these antioxidants after being subjected to animal fluids (e.g., gastric juice with acidic pH); their degree of absorption by the gastric epithelium; and the pharmacokinetics and pharmacodynamics of these compounds, among other important factors to be considered [80]. For these reasons, several researchers have focused on improving the drug or antioxidant delivery for therapeutic purposes [81]. All these points are pivotal to developing better in vivo models in the future or adapting well-defined in vivo models for a more reliable study of antioxidants and their role as potential preventive agents of *H. pylori* infection and, consequently, of the development of gastric cancer. 

## 5. Conclusions

We studied the anticancer effects of aqueous extracts of blueberry plants untreated and treated with MeJA on gastric cancer in relation to their antioxidant properties. Our findings demonstrated that blueberry leaf aqueous extracts showed higher inhibitory effects on the viability of gastric cancer cells (AGS line) than fruit extracts. Furthermore, MeJA application on blueberry plants increased antioxidant activity and compounds, mainly with respect to anthocyanins. It is noteworthy that leaf extracts from plants treated with MeJA displayed higher inhibitory effects on viability and migration capacity of AGS cells compared to extracts from MeJA-untreated plants. Furthermore, extracts of leaves from MeJA-treated plants were capable of decreasing the expression of proteins (e.g., p-P70S6K and p-ERK1/2) that induce carcinogenic properties in gastric cancer cell lines. Our outcomes also revealed that the anthocyanin-rich fraction of blueberry leaf extracts showed greater in vitro anticancer effects than the crude leaf extracts, which could be explained by the well-documented remarkable antioxidant and healthy properties of anthocyanin molecules. In spite of these promising findings, it is still unclear whether the anthocyanin-rich leaf extracts of blueberry plants are capable of exerting a chemopreventive or chemoprotective effect against gastric cancer on in vivo conditions, as there were no differences in gastric cancer protection or development between gerbils fed and unfed with blueberry extracts. In this regard, we suggest that the antioxidants in blueberry leaf extracts were poorly bioavailable for gerbils. Finally, in order to clarify the relationship between antioxidant and anticancer properties of blueberry leaf extracts, further studies are needed to more deeply investigate the mechanisms involved in the inhibition of AGS cell viability and migration as well as the effect of blueberry leaf extracts on the ROS formation in human cancer cells.

## Figures and Tables

**Table 1:** Primary antibodies used for Western blot analysis. MAPK: mitogen-activating protein kinase.

Primary Antibodies	Clone	Molecular Weight (kDa)	Dilution
mTOR	7C10	289	1/1000
AKT (pan)	11E7	60	1/1000
phospho-P70S6K(Thr389)	108D2	70, 85	1/1000
p44/42 MAPK (Erk1/2)	137F5	42–44	1/1000
Phospho-p44/42 MAPK (Erk1/2)	197G2	42–44	1/1000
β-Actin	13E5	45	1/5000

**Table 2:** Antioxidant properties of leaves aqueous extracts from blueberry plants untreated and treated with MeJA.

Crude Extract (µg mL^−1^)	Antioxidant Activity (µg TE g^−1^ FW)		Total Phenols (mg CAE g^−1^ FW)		Total Flavonoids (mg Rutin g^−1^ FW)		Total Anthocyanins (mg c3g g^−1^ FW)	
	*MeJA* *Untreated Plants*	*MeJA* *Treated Plants*	*MeJA* *Untreated Plants*	*MeJA* *Treated Plants*	*MeJA* *Untreated Plants*	*MeJA* *Treated Plants*	*MeJA* *Untreated Plants*	*MeJA* *Treated Plants*
25	30 a	90 a*	0.3 a	0.6 a*	n.d.	n.d.	2.0 a	2.1 a
50	118 b	194 b*	0.4 a	0.7 a*	0.3 a	0.4 a*	3.9 b	4.1 b
100	266 c	375 c*	0.7 b	0.9 b*	0.5 b	0.6 b	6.2 c	8.4 c*
200	569 d	748 d*	1.4 c	1.6 c	0.9 c	1.2 c*	12 d	18 d*
400	946 e	1345 e*	3.4 d	3.4 d	1.8 d	2.0 d	20 e	32 e*
800	1486 f	1733 f*	5.5 e	8.3 e*	3.9 e	4.4 e*	63 f	74 f*
1600	1924 g	1969 g*	12 f	15 f*	7.9 f	9.3 f*	88 g	127 g*
3200	1990 h	1998 h	24 g	27 g	16 g	18 g*	185 h	250 h*

Abbreviations: MeJA: methyl jasmonate; TE: Trolox equivalents; CAE: chlorogenic acid equivalents; c3g: cyanidin-3-glucoside; n.d = non-detected. Different lower case letters (a–h) indicate statistically significant differences among doses of leaf extract and asterisks (*) indicates statistical differences between MeJA-untreated and MeJA-treated extracts.

**Table 3:** Phenolic acid and flavonoid composition (analyzed by HPLC-DAD) of leaves aqueous extracts from blueberry plants untreated and treated with MeJA.

Phenolic Acids Concentration (mg g^−1^ FW)								
Crude Extract (µg mL^−1^)	Chlorogenic Acid		Cafeic Acid		Coumaric Acid		Feluric Acid	
	*MeJA* *Untreated Plants*	*MeJA* *Treated Plants*	*MeJA* *Untreated Plants*	*MeJA* *Treated Plants*	*MeJA* *Untreated Plants*	*MeJA* *Treated Plants*	*MeJA* *Untreated Plants*	*MeJA* *Treated Plants*
100	n.d.	n.d.	n.d.	n.d.	n.d.	0.27 **a**	n.d.	0.22 **a**
200	0.11 **a**	n.d.	n.d.	n.d.	0.42 **a**	0.39 **b**	0.36 **a**	0.36 **b**
400	0.53 **b**	n.d.	n.d.	n.d.	0.50 **a**	0.59 **c**	0.46 **b**	0.42 **c**
800	0.69 c*	0.26 **a**	0.54 **a**	n.d.	1.28 **b**	1.12 **d**	1.06 c*	0.88 **d**
1600	3.10 d*	0.85 **b**	1.63 b*	0.95 **a**	2.63 c*	2.31 **e**	2.27 **d**	2.04 **e**
3200	9.55 e*	5.76 **c**	4.97 c*	2.32 **b**	5.02 **d**	5.35 **f**	3.77 **e**	3.77 **f**
**Flavonoids Concentration (mg g^−1^ FW)**								
**Crude Extract** **(µg mL^−1^)**	**Rutin**		**Kaempferol-3-O**		**Myrecitin**			
	*MeJA* *untreated plants*	*MeJA* *treated plants*	*MeJA* *untreated plants*	*MeJA* *treated plants*	*MeJA* *untreated plants*	*MeJA* *treated plants*		
100	0.18 **a**	0.33 a*	n.d.	n.d.	0.67 **a**	0.76 a*		
200	0.43 **b**	0.48 **b**	n.d.	n.d.	1.00 **b**	1.21 b*		
400	0.48 **b**	0.58 **c**	n.d.	n.d.	1.66 **c**	2.13 c*		
800	0.94 **c**	1.03 **d**	1.58 **a**	1.43 **a**	2.60 **d**	3.83 d*		
1600	1.56 **d**	1.86 e*	3.09 **b**	3.04 **b**	2.54 d	5.67 e*		
3200	2.45 **e**	3.15 f*	5.70 **c**	5.83 **c**	2.70 **d**	6.91 f*		

Abbreviations: MeJA: methyl jasmonate; n.d: non-detected. Different lower case letters (a–f) indicate statistical significant differences among doses of leaves extract and asterisk (*) indicate statistical differences between control and MeJA-treated extract.

**Table 4:** Anthocyanidin composition (determined by HPLC-DAD) of leaf crude extracts from blueberry plants untreated and treated with MeJA.

Crude Extract (mg mL^-1^)	Anthocyanidin Concentration (mg g^−1^ FW)									
	Delphinidin		Malvidin		Cyanidin		Petunidin		Peonidin	
	*MeJA* *Untreated Plants*	*MeJA* *Treated Plants*	*MeJA* *Untreated Plants*	*MeJA* *Treated Plants*	*MeJA* *Untreated Plants*	*MeJA* *Treated Plants*	*MeJA* *Untreated Plants*	*MeJA* *Treated Plants*	*MeJA* *Untreated Plants*	*MeJA* *Treated Plants*
50	1.1 **a**	4.4 a*	0.25 **a**	0.54 a*	0.05 **a**	0.08 a*	n.d.	n.d.	n.d.	n.d.
100	1.5 **a**	4.9 a*	0.36 b	0.61 b*	0.12 **b**	0.16 b*	n.d.	n.d.	n.d.	n.d.
200	6.5 **b**	11 b*	0.96 c	1.2 c	0.19 **c**	0.32 c*	n.d.	n.d.	n.d.	n.d.
400	15 **c**	21 c*	2 d	2.4 d	0.39 **d**	0.66 d*	n.d.	0.09 a*	n.d.	n.d.
800	31 **d**	50 d*	3.5 e	5.1 e*	0.79 e	1.5 e*	0.08 **a**	0.14 a*	0.06 **a**	0.05 **a**
1600	59 **e**	84 e*	6.6 f	8.8 f*	1.9 f	2.8 f*	0.16 **b**	0.27 b*	0.08 **b**	0.14 b*
3200	112 **f**	153 f*	16 g	21 g*	3.6 g	5.3 g*	0.32 **c**	0.48 c*	0.20 **c**	0.24 c*

Abbreviations: MeJA: methyl jasmonate; n.d: non-detected. Different lower case letters (a–g) indicate statistically significant differences among doses of leaf extract and asterisks (*) indicates statistical differences between MeJA untreated and MeJA-treated extracts.

**Table 5:** Phenolic composition (determined by HPLC/MS/MS) of leaf crude extracts from blueberry plants untreated and treated with MeJA.

Compound	Leaves Crude Extracts	
	*MeJA Untreated Plants*	*MeJA Treated Plants*
Isocitric Cafeoil Acid	+	+
Myricetin glycoside	+	+
Kaempferol	+	+
Quercetin	+	+
Kineic Cafeoyl Acid	+	+
Quercetin-3- Glycoside	+	+
Malonil Acid Mono Cafeoil kineic	+	+
Myricetin-3-Glycoside	+	+
Myricetin-3-Xylose	+	−
Rutin	+	+
Quercetin-3 Oβ- Xyloside	+	+
Quercetin-3-Ramnoside	+	+
Kaempferol-3-O-Rutinoside	+	+
Isorhamnetine-3-Rutinoside	+	+
Quercetin-3-O-glucose	+	+

## Supplementary Materials

The following are available online at https://www.mdpi.com/2076-3921/9/1/45/s1, Table S1: Antioxidant activity, total phenols, and total anthocyanins in leaves and fruit extracts of blueberry plants (untreated with MeJA).
