# Supplementary material for: The Anti-Proliferative and Anti-Invasive Effect of Leaf Extracts of Blueberry Plants Treated with Methyl Jasmonate on Human Gastric Cancer In Vitro Is Related to Their Antioxidant Properties
Source: Antioxidants (Basel). 2020 Jan 4;9(1):45. doi: 10.3390/antiox9010045 (PMC7023271; doi:10.3390/antiox9010045)
Supplement: Supplementary file 1 [file antioxidants-09-00045-s001.pdf]

Table S1. Antioxidant activity, total phenols, and total anthocyanins in leaves and fruit extracts of blueberry plants. Different lower case indicates statistically significant differences between doses and asterisk (*) indicates statistically significant differences between extracts.

	**Doses (µg mL^{-1})**	**Antioxidant Activity (µg TE g^{-1} FW)**	**Total Phenols (µg CAE g^{-1} FW)**	**Total Anthocyanins (µg c3g g^{-1} FW)**
Fruit	100	232 ± 25b	0.40 ± 0.02b	6.6 ± 0.5b
	3200	4377 ± 95a	16.5 ± 0.81a	126 ± 1.3a
Leaves	100	4111 ± 42b*	5.1 ± 0.12b*	19 ± 0.3b*
	3200	9340 ± 19a*	28.4 ± 0.36a*	453 ± 15a*

TE: trolox equivalents; CAE: chlorogenic acid equivalents; c3g: cyanidin-3-glucoside. Different lower case indicates statistically significant differences between doses and asterisk (*) indicates statistically significant differences between extracts.
